# Clinical and Genetic Characteristics of Senior-Loken Syndrome Patients in Korea

**DOI:** 10.3390/genes16070835

**Published:** 2025-07-17

**Authors:** Jae Ryong Song, Sangwon Jung, Kwangsic Joo, Hoon Il Choi, Yoon Jeon Kim, Se Joon Woo

**Affiliations:** 1 Department of Ophthalmology, Seoul National University College of Medicine, Seoul National University Bundang Hospital, Seongnam 136705, Republic of Korea; songhi12345@snu.ac.kr (J.R.S.); namooj@snu.ac.kr (K.J.); 2 Department of Ophthalmology, Seoul National University College of Medicine, Seoul National University Hospital, Seoul 03080, Republic of Korea; lasolar2001@naver.com; 3 Department of Ophthalmology, Asan Medical Center, University of Ulsan College of Medicine, Seoul 05505, Republic of Korea; ra02891@amc.seoul.kr

**Keywords:** Senior-Loken syndrome, *NPHP1*, *NPHP4*, *IQCB1*, *SDCCAG8*

## Abstract

**Background/Objectives**: Senior-Loken syndrome (SLS) is a rare autosomal recessive renal–retinal disease caused by mutations in 10 genes. This study aimed to review the ophthalmic findings, renal function, and genotypes of Korean SLS cases. **Methods**: We retrospectively reviewed 17 genetically confirmed SLS patients in Korea, including 9 newly identified cases and 8 previously reported. Comprehensive ophthalmologic evaluations and renal assessments were conducted. Genetic testing was performed using whole-genome sequencing (WGS), whole-exome sequencing (WES), or Sanger sequencing. **Results**: Among the 17 patients, patients with *NPHP1* mutations were most common (35.3%), followed by those with *NPHP4* (29.4%), *IQCB1* (*NPHP5*, 29.4%), and *SDCCAG8* (*NPHP10*, 5.9%) mutations. Patients with *NPHP1* mutations showed retinitis pigmentosa (RP) sine pigmento and preserved central vision independent of renal deterioration. Patients with *NPHP4* mutations showed early renal dysfunction. Two patients aged under 20 maintained relatively good visual function, but older individuals progressed to severe retinopathy. Patients with *IQCB1* mutations were generally prone to early and severe retinal degeneration, typically manifesting as Leber congenital amaurosis (LCA) (three patients), while two patients exhibited milder RP sine pigmento with preserved central vision. Notably, two out of five (40.0%) maintained normal renal function at the time of diagnosis, and both had large deletions in *IQCB1*. The patient with *SDCCAG8* mutation exhibited both end-stage renal disease and congenital blindness due to LCA. Wide-field fundus autofluorescence (AF) revealed perifoveal and peripapillary hypoAF with a perifoveal hyperAF in younger patients across genotypes. Patients under 20 years old showed relatively preserved central vision, regardless of the underlying genetic mutation. **Conclusions**: The clinical manifestation of renal and ocular impairment demonstrated heterogeneity among Korean SLS patients according to causative genes, and the severity of renal dysfunction and visual decline was not correlated. Therefore, simultaneous comprehensive evaluations of both renal and ocular function should be performed at the initial diagnosis to guide timely intervention and optimize long-term outcomes.

## 1. Introduction

Senior-Loken syndrome (SLS) is a rare autosomal recessive disease characterized by nephronophthisis (NPHP, OMIM #256100) and retinal degeneration [1]. It usually progresses to end-stage renal disease requiring kidney transplantation as well as severe visual decline before the age of 20 [2]. The prevalence of NPHP is 1:1,000,000, and only about 200 cases of SLS have been reported worldwide (https://rarediseases.org, accessed on 1 March 2025), underscoring its rarity and the limited clinical understanding of the disease [1,3].

**Figure 1:**
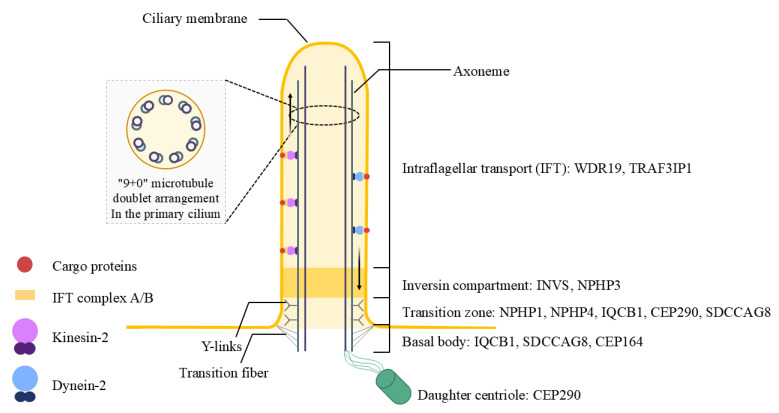
Structural domains of the primary cilium and associated genes in Senior-Loken syndrome. This diagram illustrates the organization of the primary cilium and its disease-relevant compartments in Senior-Løken syndrome. The axoneme, composed of a 9 + 0 microtubule doublet, supports bidirectional intraflagellar transport (IFT) via kinesin-2 (anterograde) and dynein-2 (retrograde) motors. The transition zone functions as a selective barrier regulating ciliary entry. Y-links connect axonemal microtubules to the ciliary membrane, and transition fibers anchor the basal body to the cell cortex. The basal body and daughter centriole serve as structural bases for ciliogenesis. Genes commonly mutated in Senior-Løken syndrome are shown according to their corresponding ciliary compartments.

To date, mutations in 10 cilia-related genes (*NPHP1, INVS/NPHP2, NPHP3, NPHP4, IQCB1/NPHP5, CEP290/NPHP6, SDCCAG8/NPHP10, WDR19/NPHP13, TRAF3IP1* and *CEP164*) have been identified in SLS patients (Figure 1) [1,4]. These genes critically influence the structural and functional integrity of primary cilia, a crucial cellular organelle in renal epithelial cells and retinal photoreceptor cells. Variability in genetic mutations leads to heterogeneous clinical presentations, making timely diagnosis and personalized treatment difficult. The retinal manifestations of SLS vary widely and include Leber congenital amaurosis (LCA), retinitis pigmentosa (RP), and tapetoretinal degeneration, further underscoring the clinical complexity of the disease [5,6].

Genotype–phenotype correlations remain poorly identified in Asian populations, including Korean SLS patients. Previous studies have briefly reported eight Korean SLS cases with either *IQCB1, NPHP1* or *SDCCAG8* gene mutations, but detailed ophthalmological and genetic evaluations are lacking [7]. Therefore, our study aims to comprehensively describe the ophthalmological characteristics and genotype–phenotype relationships of 17 Korean SLS cases, including 9 newly identified patients, to facilitate early diagnosis and guide individualized clinical management strategies.

## 2. Materials and Methods

### 2.1. Patients and Clinical Data Collection

This study was conducted in accordance with the Declaration of Helsinki and approved by the Institutional Review Boards of Seoul National University Bundang Hospital (SNUBH) (IRB No: B-2307-843-104) and Asan Medical Center (AMC) (IRB No: S2025-1000-0002). Informed consent was obtained from all participants prior to genetic testing. We retrospectively reviewed the medical records of 17 patients with genetically confirmed Senior-Loken syndrome (SLS), including 9 newly diagnosed cases and 8 previously published Korean cases. Of the 9 new cases, 5 (from 3 unrelated families) were evaluated at SNUBH and 4 (from 4 unrelated families) at Asan Medical Center between January 2015 and October 2023. Eight previously reported Korean SLS cases (reported before December 2023) were identified through PubMed search, and their detailed genetic and clinical information was reviewed. Patients with other syndromic ciliopathies (e.g., Bardet-Biedl syndrome, Joubert syndrome, Jeune syndrome, Meckel syndrome) were excluded to specifically isolate Senior-Loken syndrome and to ensure precise characterization of its unique clinical and genetic features.

SLS was diagnosed through a complete ophthalmic examination, renal ultrasonography, renal function test, abdominal/brain radiologic examination, and genetic workup. Clinical data included age, sex, symptoms at initial presentation, final best-corrected visual acuities (BCVAs), refractive error, slit lamp examination, and detailed fundus examination using ultrawide-field (UWF) fundus photography, UWF fundus autofluorescence, spectral-domain optical coherence tomography (SD-OCT; Spectralis OCT; Heidelberg Engineering, Heidelberg, Germany). Retinal function was evaluated via full-field electroretinography (ff-ERG) using the protocol recommended by the International Society for Clinical Electrophysiology of Vision (ISCEV) [8,9,10,11], and visual fields were assessed via Goldmann visual field (GVF) or Humphrey visual field (HVF) testing. Renal function was evaluated through urinalysis, serum creatinine, and blood urea nitrogen.

### 2.2. Genotyping

Patients were genotyped using whole-genome sequencing (WGS), whole-exome sequencing (WES) or direct (Sanger) sequencing, with each institution following its established methods. For patients examined at SNUBH, genetic data were processed by 3billion (https://3billion.io/index, accessed on 1 March 2025). Genomic DNA was extracted from whole blood samples collected from peripheral blood. For WGS, sequencing libraries were prepared using the TruSeq DNA PCR-Free kit (Illumina, San Diego, CA, USA), while for WES, exome capture employed the xGen Exome Research Panel v2 (Integrated DNA Technologies, Coralville, Iowa, USA). Both WGS and WES were performed on the NovaSeq X system (Illumina, San Diego, CA, USA). The generated sequence data were aligned to the Genome Reference Consortium Human Build 38 (GRCh38) and the Revised Cambridge Reference Sequence (rCRS) for human mitochondrial DNA. Sequencing data analysis and variant interpretation utilized the EVIDENCE v4.2 automated variant analysis system developed by 3billion [12].

For single-nucleotide variant (SNV)/small insertion/deletion (INDEL) detection, the Genome Analysis Toolkit (GATK) best practices were employed [13]. Copy number variants (CNVs) and structural variants (SVs), including aneuploidy, were detected using custom programs 3bCNV v23.0818 and Manta with depth of coverage (DOC) information [14]. Low-level heteroplasmic SNV/INDEL detection in the mitochondrial genome used Mutect2, while repeat expansion detection employed ExpansionHunter v5.0.0, and mobile element insertion detection utilized MELT v2.2.2 [13,15,16]. Regions of homozygosity (ROH) were identified using AutoMap v1.2, and variant annotation was performed with Variant Effect Predictor v104.2 [17,18]. Variant selection and classification followed the guidelines recommended by the American College of Medical Genetics and Genomics (ACMG) and the Association for Molecular Pathology (AMP) [19,20,21].

For direct (Sanger) sequencing, PCR primers were designed using Primer3 (v. 0.4.0), Whitehead Institute (http://bioinfo.ut.ee/primer3-0.4.0/, accessed on 1 March 2025), and NCBI GenBank reference sequences [22,23]. PCR amplification and Sanger sequencing were conducted using the PCR Master Mix Kit, ExoSAP-IT^TM^ PCR Product Cleanup Reagent, BigDye^TM^ Terminator v3.1 Cycle Sequencing Kit (all from ThermoFisher Scientific, Waltham, MA, USA), and SeqStudio Genetic Analyzer (Applied Biosystems, Foster City, CA, USA). Sequencing results were analyzed using Sequence Scanner Version 2.0 (Applied Biosystems, Foster City, CA, USA).

### 2.3. Statistical Analysis

All analyses were performed using IBM SPSS Statistics version 19.0 (IBM Corp., Armonk, NY, USA). Comparisons between groups were made using the Kruskal–Wallis test. A *p*-value of <0.05 was considered statistically significant.

## 3. Results

### 3.1. Demographic and Baseline Clinical Characteristics

A total of 17 patients with genetically confirmed SLS, including 9 newly identified and 8 previously reported cases, were analyzed. The mean age at diagnosis was 18.3 ± 11.4 years, ranging from 8.1 to 49 years, and 47.1% of patients were female. Excluding previously reported cases, the mean log MAR BCVA at the last visit was 0.44 ± 0.55. Although high hyperopia has been typically observed in SLS, particularly in *IQCB1*-related cases [6,24,25,26], the mean spherical equivalent in our cohort was −1.20 ± 2.34 D, with most patients showing myopia, regardless of genotype. Only one patient with the *IQCB1* mutation showed hyperopia (+1.25 D S.E.). At the time of diagnosis, 76.5% (13/17) of patients had progressed to end-stage renal disease (ESRD) or had undergone kidney transplantation, 11.8% (2/17) had moderate chronic kidney disease and 11.8% (2/17) had normal renal function. Detailed clinical and genetic characteristics of all 17 patients, including genotype, visual function and renal status, are shown in Table 1 and Table 2.

### 3.2. Clinical Characteristics by Genotype

The *NPHP1*-related SLS was the most common (35.3%, 6/17), while *NPHP4*-related SLS, *IQCB1*-related SLS, and *SDCCAG8*-related SLS accounted for 29.4% (5/17), 29.4% (5/17) and 5.9% (1/17), respectively. Patients with *NPHP1* mutations (*n* = 6, 35.3%) typically demonstrated RP sine pigmento or retinal dystrophy, maintaining relatively preserved central vision independent of renal deterioration. These patients commonly exhibited central visual preservation and moderate retinal degeneration, with generally preserved ERG responses initially that progressed with age. Among the five patients diagnosed with *NPHP4*-related SLS, two patients younger than 20 years old had moderate chronic kidney disease and ESRD, respectively, but showed normal visual fields and either normal or decreased cone response on the ff-ERG. On the other hand, the three patients over the age of 30 years old had progressed to ESRD or had undergone kidney transplantation and showed severe retinopathy with no ff-ERG responses, reflecting more advanced retinal degeneration. Patients with *IQCB1* (*NPHP5*) mutations (*n* = 5, 29.4%) typically displayed early and severe retinal degeneration, characterized by congenital blindness due to Leber congenital amaurosis (LCA) in three cases. Notably, two sibling patients presented with RP sine pigmento and preserved central vision, maintaining normal renal function at diagnosis. These two cases had large deletions in *IQCB1* identified via WGS, a finding previously unreported using conventional methods [7]. The patient with an *SDCCAG8* (*NPHP10*) mutation exhibited severe dual-organ involvement with end-stage renal disease and congenital blindness due to LCA. A summary of subgroup characteristics by gene mutation is provided in Table 3. In our study, all SLS patients showed bilaterally similar findings in fundus examination, ff-ERG, visual field, and last BCVA.

### 3.3. Age-Related Phenotypic Patterns

**Figure 2:**
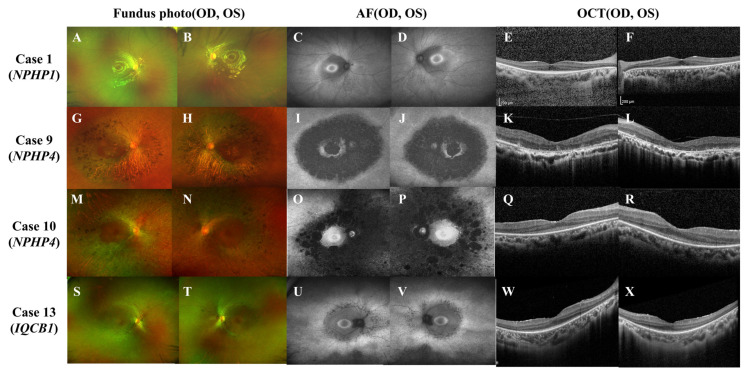
Representative ophthalmic findings of Case 1, 9, 10, and 12. In case 1 (**A**–**F**, *NPHP1*: homozygous whole gene deletion), wide-field fundus photographs (WFP; **A**,**B**) show classic retinitis pigmentosa (RP) sine pigmento with a preserved fovea. Spectral-domain optical coherence tomography (OCT; **E**,**F**) reveals parafoveal photoreceptor (PR) loss sparing the foveal center. Wide-field fundus autofluorescence (WFAF; **C**,**D**) demonstrates a perifoveal hypo-autofluorescent ring bordered by a hyper-autofluorescent bull’s-eye pattern. In case 9 (**G**–**L**, *NPHP4*: c.1972C > T; c.453-1G > C, compound heterozygous), WFP (**G**,**H**) depicts central and pericentral RP with coarse bone-spicule pigmentation. OCT (**K**,**L**) shows bilateral perifoveal PR degeneration. WFAF (**I**,**J**) displays diffuse perifoveal hypo-autofluorescence encircled by a hyper-autofluorescent ring. In case 10 (**M**–**R**, *NPHP4*: c.2964del, homozygous), WFP (**M**,**N**) demonstrates bilateral central-sparing diffuse RP and peripheral pigmentation. OCT (**Q**,**R**) shows widespread outer-retinal atrophy sparing the foveal center. WFAF (**O**,**P**) highlights marked perifoveal and peripapillary hypo-autofluorescence with foveal sparing. In Case 12 (**S**–**X**, *IQCB1*: c.1522_1523dup; 5.8-kb intronic deletion), WFP (**S**,**T**) reveals RP sine pigmento without overt pigment migration. OCT (**U**,**V**) exhibits parafoveal PR disruption. WFAF (**W**,**X**) shows a hyper-autofluorescent bull’s eye ring surrounded by perifoveal hypo-autofluorescence.

Regardless of genotype, younger patients under 20 years old generally preserved central vision (mean logMAR BCVA of 0.16 ± 0.27) despite mild visual-field defects. Fundus photography revealed atypical RP sine pigmento features, while wide-field autofluorescence imaging showed perifoveal and peripapillary hypo-autofluorescence with a hyper-autofluorescent bull’s eye-like ring (Figure 2, Case 1 and 12). Conversely, patients aged over 30 displayed typical RP findings, such as bony spicules, severe visual impairment, and significant ERG abnormalities, alongside frequent ESRD (Figure 2, Case 9 and 10).

## 4. Discussion

In our cohort of 17 Korean patients with genetically confirmed Senior-Loken syndrome (SLS), four causative genes were identified: *NPHP1* (35.3%), *NPHP4* (29.4%), *IQCB1* (29.4%), and *SDCCAG8* (5.9%). Patients with *NPHP1* mutations typically showed RP sine pigmento and retained central vision despite significant renal decline. In contrast, *IQCB1*-related cases exhibited early-onset retinal degeneration, frequently manifesting as LCA, but some retained normal renal function at diagnosis. NPHP4 mutations were associated with early kidney impairment and variable retinal findings depending on age, while the single *SDCCAG8* case presented with severe dual-organ involvement. Notably, large homozygous deletions in *NPHP1* were the most frequent variant type (23.5%) and would likely have been missed by conventional sequencing, emphasizing the importance of genome-wide analysis in suspected SLS.

SLS is a genetically heterogeneous renal–retinal ciliopathy associated with multiple ciliopathy-related genes, including *NPHP1*, *NPHP4*, *IQCB1*, and *SDCCAG8*. Unlike syndromic ciliopathies such as Bardet-Biedl, Joubert, or Meckel syndromes, which involve multiple organ systems, SLS primarily affects the kidney and retina [1]. These two organs share developmental and structural similarities, including dependence on collagen IV and primary cilia function [27].

Our study extends the previous literature, indicating variability in gene prevalence among SLS cohorts. Large deletions were identified in 47% (8/17) of our cohort, predominantly affecting NPHP1 (six cases) and NPHP4 (two cases). Our study extends the previous literature, indicating variability in gene prevalence among SLS cohorts, with notably higher frequencies than previously reported. Hildebrandt et al. found *NPHP1* mutations in 21% and *IQCB1* in 3% of nephronophthisis families [4], while Wang et al. reported *CEP290* as the most frequent gene (42.7%) in 1301 SLS-affected families, followed by *NPHP1* (17.4%) and *IQCB1* (13.3%) [28]. However, our Korean cohort showed striking differences: *NPHP1* mutations were found in 35.3% of patients (vs. 17.4–21% in international studies), with homozygous deletions representing the single most frequent variant type. Furthermore, the IQCB1 c.1522_1523dup variant was identified in five patients from three unrelated families (29.4% of our cohort), compared to only 3–13.3% reported globally [4,28], suggesting a founder mutation in the Korean population. Additionally, our cohort had no *CEP290* mutations despite comprehensive screening, contrasting sharply with the 42.7% frequency reported by Wang et al. [28]. These findings demonstrate significant population-specific mutation patterns that extend beyond simple racial or geographic variation, indicating distinct genetic architecture in Korean SLS patients. These population-specific findings have direct clinical implications: targeted screening for *NPHP1* deletions and *IQCB1* c.1522_1523dup in Korean individuals with compatible phenotypes could expedite diagnosis and inform genetic counseling. The absence of *CEP290* mutations suggests that Korean patients may not require prioritized screening for this gene, allowing resources to be focused on population-relevant variants. This emphasizes the need for population-specific profiling and tailored diagnostic strategies to achieve optimal detection rates in Korean SLS patients.

Furthermore, our findings revealed distinct genotype–phenotype correlations. In our study, *NPHP1*-related patients typically exhibited RP sine pigmento and maintained central vision, although retinal function declined with age. *NPHP4*-mutated cases typically had progressed renal disease at diagnosis, with variable ocular involvement depending on age. *IQCB1* mutations were associated with early-onset retinal degeneration, including LCA, though some patients had preserved renal function. The *SDCCAG8*-related case showed severe dual-organ involvement with both ESRD and congenital blindness. These patterns are consistent with previous studies [4,28]. Notably, central vision tended to be preserved in late-onset retinopathy regardless of genotype, indicating that retinal and renal severities are not always aligned.

These genotype–phenotype correlations are supported by the biological roles of the corresponding genes. *NPHP1* encodes nephrocystin-1, a transition zone protein critical for intraflagellar transport [1,29,30]. Although its loss impairs the trafficking of proteins into the cilium, the axonemal structure often remains intact, which may explain the relatively mild retinal phenotype, such as RP sine pigmento, observed in our *NPHP1*-related patients (Cases 1 and 2), despite their rapid progression to ESRD (Cases 1, 3–6). In contrast, *NPHP4*, which interacts with *NPHP1* and regulates its localization via Pyk2-mediated phosphorylation, is more directly involved in photoreceptor maintenance. This aligns with our *NPHP4*-related cases (Cases 9–11), where older patients exhibited severe diffuse RP with extinguished ERG responses and renal failure, while younger patients (Cases 7 and 8) showed preserved visual function and milder retinal involvement [30,31,32,33,34].

*IQCB1*, also known as *NPHP5*, plays a critical role in the formation of the ciliary transition zone in photoreceptors [6]. Its deficiency leads to early disruption of outer-segment development [35], explaining the early-onset RP or LCA observed in all of our *IQCB1*-related patients (Cases 12–16). However, since renal cilia may remain structurally intact for a longer period, two of our patients (Cases 12 and 13) maintained preserved renal function at diagnosis [35,36]. Lastly, *SDCCAG8*, which co-localizes with IQCB1 and RPGRIP1 at the transition zone, is implicated in both retinal and renal ciliopathy [37]. The coexistence of LCA and ESRD in our *SDCCAG8*-related patient (Case 17) supports the pathogenic role of this complex in SLS [31]. Collectively, the differential organ involvement pattern depending on the affected gene indicates that SLS progression is not a simple retina-first or kidney-first paradigm. Rather, it reflects gene-specific and environment-specific vulnerabilities, including tissue-specific susceptibility governed by differential gene expression levels, reliance on distinct ciliary protein complexes, and exposure to organ-specific stressors. These factors necessitate independent and tailored surveillance strategies for each organ system.

In addition, WGS plays a critical role in detecting structural variants that are often missed by conventional methods. In particular, large deletions in *NPHP1* and *IQCB1* may be missed by WES or targeted panels. In our cohort, large deletions including the homozygous deletions in *NPHP1* were the most common variant type, accounting for 23.5% (4/17) of all cases. WGS was essential for the identification of these variants. In support of this, a recent Korean national study demonstrated the utility of WGS in inherited retinal diseases by identifying large deletions in *IQCB1* and *PRPF31* that were missed by WES or panel testing [38]. This highlights the importance of incorporating WGS into the diagnostic workflow for suspected ciliopathy-related syndromes.

In the previous literature, various retinal features such as typical RP, sector RP, RP sine pigmento and LCA have been reported in SLS patients. *NPHP1*-related SLS (c.555dup) showed macular atrophy and far peripheral retinal degeneration, while sector RP findings were observed in two individuals of Arab or Jewish descent with *NPHP1*-related SLS (deletion, exon 14–15 deletion, respectively) [28,39]. Ning K et al. reported a case of RP sine pigmento findings in *NPHP1*-related SLS (deletion) [29]. Similarly to the case reported by Ning K et al., both of our *NPHP1*-related SLS patients (deletion) showed RP sine pigmento. Additionally, our two *IQCB1*-related SLS patients (c.1522_1523dup/delin) also exhibited RP sine pigmento, similar to the three *IQCB1*-related SLS cases (c.1090C > T) reported by Wang et al. [28].

For the prognosis of SLS, early diagnosis is critical for preserving renal function and planning transplantation. According to the 2006 NAPRTCS database review, early living donor (LD) kidney transplantation in SLS patients was associated with favorable outcomes, including significantly improved long-term graft survival and slower decline in renal function compared to the general pediatric transplant population [40]. However, two of our *NPHP4* patients progressed to ESRD despite previous transplants, suggesting that post-transplant prognosis may still vary by genotype, highlighting the need for close and personalized follow-up.

There are several limitations in our study. First, the sample size was small, making it difficult to derive statistically significant results. Second, due to the retrospective nature of this study, it was challenging to establish relationships between gene mutation types, patterns of retinal degeneration, and renal function over time. Additionally, data from our case series were combined with those retrieved from previous studies. Additionally, our cross-sectional design limits the interpretation of autofluorescence findings, as we could not assess temporal dynamics or establish predictive relationships between AF patterns and disease progression. Longitudinal studies would be valuable to determine the prognostic significance of the hyper-autofluorescence patterns observed in younger patients. Nevertheless, this study presents the first comprehensive Korean case series of SLS patients that integrates ophthalmologic, genetic, and nephrologic data. It provides a valuable background for understanding the genotype-related clinical diversity and for establishing gene-specific diagnostic and management strategies.

## 5. Conclusions

In conclusion, this study reveals that Korean patients with Senior-Loken syndrome display distinct renal and ocular phenotypes based on the specific causative gene. Notably, there was no consistent correlation between the severity of kidney and retinal involvement. Therefore, once SLS is suspected or diagnosed, the comprehensive and concurrent evaluation of both renal and ocular function is essential to ensure accurate diagnosis and appropriate clinical management. Furthermore, large deletions, especially in *NPHP1* and *IQCB1*, may be missed by panel testing or WES, making WGS a critical tool for accurate diagnosis. Early identification through WGS and timely, gene-specific clinical decision making are critical to improve long-term outcomes for both eye and kidney function in patients with SLS.

## Figures and Tables

**Table 1:** Genetic characteristics of Korean Senior-Loken syndrome patients.

Case	Gene	GenBank Accession	Coding Sequence Variant	Zygosity	Amino Acid Change	Reference
1	*NPHP1*	NM_000272.5	Whole gene deletion	Homozygous	p.?	Novel
2	*NPHP1*	NM_000272.5	c.143G > A; c.1578_1582delinsG	Heterozygous	p.Arg48Lys; p.(Asp526Glufs*16)	Novel
3	*NPHP1*	NM_000272.5	Whole gene deletion	Homozygous	p.?	[7]
4	*NPHP1*	NM_000272.5	Whole gene deletion	Homozygous	p.?	[7]
5	*NPHP1*	NM_000272.5	Whole gene deletion	Homozygous	p.?	[7]
6	*NPHP1*	NM_000272.5	c.609_610insC; Whole gene deletion	Heterozygous	p.Arg204Glnfs*8; p.?	[7]
7	*NPHP4*	NM_015102.5	c.2304 + 1G > A; c.280_673del (exons 4–6)	Heterozygous	p.?; p.(Pro94Alafs*90)	Novel
8	*NPHP4*	NM_015102.5	c.2304 + 1G > A; c.280_673del (exons 4–6)	Heterozygous	p.?; p.(Pro94Alafs*90)	Novel
9	*NPHP4*	NM_015102.5	c.1972C > T; c.453-1G > C	Heterozygous	p.Arg658*; p.?	Novel
10	*NPHP4*	NM_015102.5	c.2964del	Homozygous	p.Glu989Serfs*17	Novel
11	*NPHP4*	NM_015102.5	c.2304 + 1G > A; c.1972C > T	Heterozygous	p.?; p.Arg658*	Novel
12	*IQCB1*	NM_001023570.4	c.1522_1523dup; 5.8 Kb intron deletion	Heterozygous	p.Ala509Lysfs*3; p.?	Novel
13	*IQCB1*	NM_001023570.4	c.1522_1523dup; 5.8 Kb intron deletion	Heterozygous	p.Ala509Lysfs*3; p.?	Novel
14	*IQCB1*	NM_001023570.4	c.1522_1523dupGA	Homozygous	p.Ala509Lysfs*3	[7]
15	*IQCB1*	NM_001023570.4	c.1522_1523dupGA	Homozygous	p.Ala509Lysfs*3	[7]
16	*IQCB1*	NM_001023570.4	c.1522_1523dupGA	Homozygous	p.Ala509Lysfs*3	[7]
17	*SDCCAG8*	NM_006642.5	c.845_848delTTTG; c.1300delA	Heterozygous	p.Cys283fs1; p.Asn434Ilefs28	[7]

* indicates stop codon.

**Table 2:** Clinical characteristics of Korean Senior-Loken syndrome patients.

Case	Age at Diagnosis (yrs)/Sex	Age at Symptom Onset (yrs)/Organ	Renal Status	Ocular Phenotype	Visual Field Pattern	Last BCVA (RE, LE)	Full Field ERG Response
1	12/F	12/Kidney	ESRD	RP sine pigmento, Strabismus	OU central 7° preserved	20/16, 20/25	No rod/cone response
2	17/M	8/Eye	Moderate CKD	RP sine pigmento	OU central and superotemporal scotoma	20/25, 20/30	Extinct rod, preserved cone
3	8/M	NA	ESRD	RP	NA	NA	NA
4	13/F	NA	ESRD	RP, nystagmus	NA	NA	NA
5	14/M	NA	ESRD	RP	NA	NA	NA
6	13/M	NA	ESRD	RP, nystagmus	NA	NA	NA
7	13/F	13/Kidney	ESRD	Fundus albifunctatus	Normal VF	20/20, 20/20	Within normal limit
8	16/F	15/Kidney	Moderate CKD	Cone dystrophy	Normal VF	20/16, 20/20	Decreased cone, preserved rod
9	49/M	20/Eye	*s*/*p* 1st KTPL age 37	Central & pericentral RP	OU central scotoma with 30–50° ring preserved	20/600, 20/600	No rod/cone response
10	31/F	23/Eye	ESRD	Diffuse RP	OU central 4° preserved	20/600, 20/120	No rod/cone response
11	39/M	12/Kidney	*s*/*p* 1st KTPL age 12, *s*/*p* 2nd KTPL age 39	Diffuse RP	OU central 8° preserved	20/50, 20/60	No rod/cone response
12	15/M	15/Eye	Normal	RP sine pigmento	OU central 3° preserved	20/100, 20/100	No rod/cone response
13	13/M	13/Eye	Normal	RP sine pigmento	NA	20/25, 20/32	NA
14	18/F	NA	ESRD	LCA	NA	NA	NA
15	11/M	NA	ESRD	LCA, nystagmus	NA	NA	NA
16	11/F	NA	ESRD	LCA	NA	NA	NA
17	14/F	NA	ESRD	LCA, strabismus	NA	NA	NA

Abbreviations: BCVA, best-corrected visual acuity; CKD, chronic kidney disease; ERG, electroretinogram; ESRD, end-stage renal disease; F, female; KTPL, kidney transplantation; LCA, Leber congenital amaurosis; LE, left eye; M, male; NA, not available; OU, both eyes; RE, right eye; RP, retinitis pigmentosa; s/p, status post; VF, visual field; yrs, years.

**Table 3:** Demographics and subgroup analysis based on gene mutation type.

Parameters	Total	Mutated Gene			*p*-Value	
		*NPHP1*	*NPHP4*	*IQCB1*	*SDCCAG8*	
		(NPHP1)	(NPHP4)	(NPHP5)	(NPHP10)	
No. of patients	17 (100%)	6 (35.3%)	5 (29.4%)	5 (29.4%)	1 (5.9%)	
Age at diagnosis, yrs	18.3 ± 11.4 (8.1–49.0)	12.8 ± 2.9 (8.1–17.0)	29.6 ± 15.2 (13–49)	13.5 ± 3.0 (10.8–17.9)	NA	0.058 *
Age at onset, yrs ^†^	14.6 ± 4.5 (8–23)	10.0 ± 2.8 (8–12)	16.6 ± 4.7 (12–23)	14.0 ± 1.4 (13–15)	NA	0.183 *
Sex (Male/Female)	9/8	4/2	2/3	3/2	0/1	0.825 **
logMAR BCVA at last follow-up ^†^	0.44 ± 0.55 (−0.10 to 1.48)	0.07 ± 0.12 (−0.10 to 0.18)	0.60 ± 0.67 (−0.10 to 1.48)	0.43 ± 0.32 (0.10 to 0.70)	NA	0.362 *
Spherical equivalent(Diopter, D) ^†^	−1.20 ± 2.34 (−5.50 to 3.00)	−2.69 ± 0.91 (−3.63 to −1.63)	−1.09 ± 2.89 (−5.50 to 3.00)	−0.06 ± 1.07 (−1.25 to 1.25)	NA	0.170 *
No. of ESRD or S/P KTPL	13/17 (76.5%)	5 (83%)	4 (80%)	3 (60%)	1 (100%)	0.794 **
No. of LCA or RP	15/17 (88.2%)	6 (100%)	3 (60%)	5 (100%)	1 (100%)	0.167 **
No. of LCA	4/17	0 (0%)	0 (%)	3 (60%)	1 (100%)	**0.036** **
VF pattern ^†^						
Normal VF	2/8 (25%)	0 (0%)	2 (40%)	0 (0%)	NA	0.258 **
Central VF defect	2/8 (25%)	1 (50%)	1 (20%)	0 (0%)	NA	0.538 **
Central VF preserved with peripheral VF constriction	4/8 (50%)	1 (50%)	2 (20%)	1 (100%)	NA	0.504 **
Full field ERG pattern ^†^						
Within normal limit	1/8 (12.5%)	0	1 (20%)	0	NA	>0.999 **
Decreased cone response	1/8 (12.5%)	0	1 (20%)	0	NA	>0.999 **
Decreased rod response	1/8 (12.5%)	1 (50%)	0 (0%)	0	NA	0.058 **
Decreased cone and rod response	5/8 (62.5%)	1 (50%)	3 (60%)	1 (100%)	NA	0.610 **
Initial symptom ^†^						
Visual symptom	5/9 (55.6%)	1 (50%)	2 (40%)	2 (100%)	NA	0.683 **
Renal symptom	4/9 (44.4%)	1 (50%)	3 (60%)	0 (0%)	NA	0.683 **
Diagnosed priorities ^†^						
Eye	5/9 (55.6%)	1 (50%)	2 (40%)	2 (100%)	NA	0.683 **
Kidney	4/9 (44.4%)	1 (50%)	3 (60%)	0 (0%)	NA	0.683 **
Family history ^†^	4/9 (44.4%)	0	2 (40%) ^‡^	2 (40%) ^‡^	NA	0.365 **

Values are presented as number, ratio (%) or mean ± standard deviation (range), unless otherwise indicated. *p*-values of < 0.05 are considered statistically significant. Significant values are in [bold]. Abbreviation: BCVA, best corrected visual acuity; ERG, electroretinogram; ESRD, end-stage renal disease; KTPL, kidney transplantation; LCA, Leber congenital amaurosis; NA, not applicable; RP, retinitis pigmentosa; S/P, status post; VF, visual field; yrs, years. * Kruskal-Wallis One-Way Analysis of Variance excluding *SDCCAG8* mutation group. ** Fisher’s exact test of Variance excluding *SDCCAG8* mutation group. ^†^ Analysis of 18 eyes in our 9 cases. ^‡^ Cases 7–8 (NPHP4 mutations) and Cases 12–13 (IQCB1 mutations) represent two separate sibling pairs from unrelated families.

## Data Availability

The data that support the findings of this study are available from the corresponding author upon reasonable request.

## Abbreviations

The following abbreviations are used in this manuscript:

BCVA	Best-Corrected Visual Acuity
ERG	Electroretinogram
ESRD	End-Stage Renal Disease
GVF	Goldmann Visual Field
LCA	Leber Congenital Amaurosis
NPHP	Nephronophthisis
OCT	Optical Coherence Tomography
RP	Retinitis Pigmentosa
SLS	Senior-Loken Syndrome
VF	Visual Field
WES	Whole-Exome Sequencing
WGS	Whole-Genome Sequencing
